# Primary extragastrointestinal stromal tumor of the prostate: review of the literature and case report

**DOI:** 10.1097/MS9.0000000000000373

**Published:** 2023-04-11

**Authors:** Wajdi Benabdallah, Mouna Ben Othmane, Ines Ouahchi, Sarra Mestiri, Oussama Belkacem, Khaireddine Bouassida, Wissem Hmida, Mehdi Jaidane

**Affiliations:** a Urology Department; b Pathology Department, Sahloul Hospital; c Department of Cytogenetic and Reproductive Biology, Farhat Hached University Teaching Hospital, Sousse, Tunisia

**Keywords:** case report, immunohistochemistry, prostate, prostatic extragastrointestinal stromal tumor

## Abstract

The extragastrointestinal stromal tumor (EGIST) is defined as a mesenchymal neoplasm arising from soft tissues outside the gastrointestinal tract, and the prostate is a rare presentation site.

**Case presentation:**

A 58-year-old man was presented with lower urinary tract symptoms for 6 months. A digital rectal examination revealed a markedly enlarged prostate with a smooth, bulging surface. Prostate-specific antigen density was 0.5 ng/ml. MRI of the prostate showed an enlarged prostatic mass with hemorrhagic necrosis. Transrectal ultrasound-guided prostate biopsy was performed and pathological reports suggested a gastrointestinal stromal tumor. The patient refused radical prostatectomy and received only imatinib treatment.

**Clinical discussion:**

The diagnosis of EGIST of the prostate is extremely rare and depends on the histopathologic features with immunohistochemical results. The treatment is essentially based on radical prostatectomy, but there are other therapeutic modalities associating surgery with adjuvant or neoadjuvant chemotherapy. For patients refusing surgery, treatment with imatinib alone appears to be a therapeutic solution.

**Conclusion:**

Despite the rarity, EGIST of the prostate should be included in the differential diagnosis of patients presenting with lower urinary tract symptoms. There is no consensus regarding the treatment of EGIST, and the patients are treated as per the risk stratification.

## Introduction

HIGHLIGHTS

- Extragastrointestinal stromal tumor (EGIST) of the prostate is exceptionally rare.
- There is no consensus regarding the treatment of EGIST of the prostate.
- Review of the literature.

Extragastrointestinal stromal tumor (EGIST) is defined as a mesenchymal neoplasm arising from soft tissues outside the gastrointestinal tract, which is morphologically, histologically, and immunophenotypically similar to its gastrointestinal counterpart. However, the prostate is a rare presentation site. EGIST of the prostate is defined as a mass in the prostate in radiologic imaging techniques. Diagnostic biopsy is essential in therapeutic approaches. The diagnosis of EGIST depends on the histopathologic features with immunohistochemical results. Indeed, immunohistochemistry has a major role in differential diagnosis. Since their Cajal and/or Cajal-like cell origin, most of these tumors express KIT (CD117) tyrosine kinase and show the presence of activating mutations in KIT or platelet-derived growth factor receptor-α^1^. EGIST of the prostate is exceptionally rare. To the best of our knowledge, only 16 cases have been reported in the literature as primary prostatic gastrointestinal stromal tumors (GISTs) (Table 1). We herein report an unusual case of prostatic EGIST diagnosed in a 58-year-old man and discuss its presentation, diagnosis, and management with a comprehensive literature review. This case report has been reported in line with the SCARE (Surgical CAse REport) Criteria^18^.

**Table 1:** Review of literature

References	Age	PSA	Size	Treatment	Outcome
Van der Aa *et al*.^2^	49	1.36	14.2	Imatinib	The mass volume was reduced after 8 weeks. Additional follow-up to 100 weeks, the patient in good condition and reduced mass volume
Lee *et al*.^3^	75	0.2	6.7	Radical prostatectomy	Observed for 6 months and is in good condition except for mild urinary incontinence
Yinghao *et al*.^4^	49	1.1	8.5	Radical prostatectomy	No recurrence or metastasis was found (14 months)
Ou *et al*.^5^	39	0.87	10	Radical prostatectomy+adjuvant imatinib	No recurrence, no metastasis (24 months)
Zhang *et al*.^6^	31	0.37	6.5	Imatinib (intermittently due to financial reasons)	Mass volume increased, urinary retention, intestinal obstruction, and death
Liu *et al*.^7^	55	2.01	10.5	Radical prostatectomy+adjuvant imatinib	No recurrence, no metastasis (12 months)
Huh *et al*.^8^	50	0.85	9.7	Patient refused radical surgery	Left the hospital
Reinke *et al*.^9^	78	Normal	10	Imatinib	Significant reduction in the size of the tumor. No evidence of tumor progression or metastatic disease (12 months)
Almagharbi *et al*.^10^	84	5.4	17	Transvesical open prostatectomy	No recurrence
Alabed *et al*.^11^	49	5.8	10	Imatinib for 6 months+radical prostatectomy	Monitoring treatment by FDG PET/CT (complete metabolic response in a residual mass)
Garg *et al*.^12^	55	3.2	Massively enlarged prostate filling almost the entire pelvic cavity	Imatinib (the patient was a known case of heart disease, and he was deemed high risk for surgical intervention)	The patient reported mild to moderate improvement in LUTS. Reduction in size of the prostatic mass (12 months)
Shen *et al*.^13^	43	2.7	13	Imatinib neoadjuvant+radical surgery (after 23 days)+imatinib adjuvant	The patient was still in good physical condition and no recurrence or distant metastasis was observed (6 months)
Schöffski *et al*.^14^	60	No PSA	12	Imatininb neoadjuvant+radical prostatectomy (after 2 years)+imatinib adjuvant	A total perioperative treatment duration of 3 years. The patient is tolerating treatment with no adverse events and no disease-specific complaints (12 months)
Li *et al*.^15^	62	1.2	9.5	Imatinib	The tumor volume decreased, no metastasis or recurrence (6 months)
Lu *et al*.^16^	65	1.41	12.5	Radical prostatocystotomy and ileal conduit+imatinib and bicalutamid adjuvant therapy for 3 months	No recurrence. Elevated PSA level were observed within 19 months during follow-up
Yang *et al*.^17^	62	1.45	9.5	Imatinib (patient refused radical prostatectomy)	At the 6-month follow-up, the prognosis was good. The symptoms of dysuria in the patient improved, and the tumor did not significantly enlarge or metastasize
Present case	58	0.5	16.5	Imatinib	2 months, in good health, without complications
Conclusion of literatue review	Average age 56.7	Normal levels of PSA <4 : 86% >4 : 13% average : 1.89	Average tumor size 11	Treatment: -Only imatinib 41.17% -Only radical prostatectomy 11.76% -Radical prostatectomy+adjuvant imatinib 11.76% -Imatinib neoadjuvant+radical prostatectomy+adjuvant imatinib 11.76% -Imatinib neoadjuvant+radical prostatectomy 5.88% -Radical prostatocystotomy and ileal conduit+imatinib and bicalutamid adjuvant 5.88% -The patient refused treatment and left the hospital 5.88% -Transvesical open prostatectomy 5.88%	The mean follow-up period was 10.3

FDG PET/CT, fluorodeoxyglucose positron emission tomography/computed tomography; LUTS, lower urinary tract symptoms; PSA, prostate-specific antigen.

## Case report

**Figure 1:**
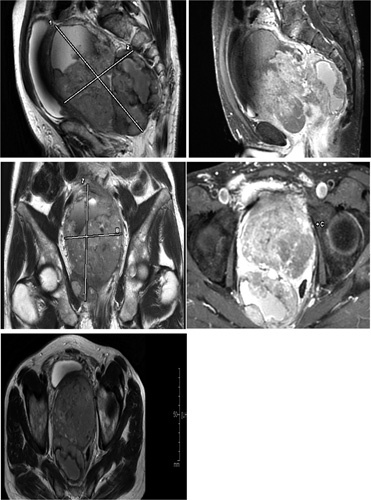
Magnetic resonance image of the prostate (our case).

A 58-year-old man presented with lower urinary tract symptoms (LUTS) that had been present for 6 months. He had a family history of a sister who died due to breast cancer at age 36. A digital rectal examination revealed a markedly enlarged prostate with a smooth, bulging surface. The serum prostate-specific antigen (PSA) density was 0.5 ng/ml. Transabdominal ultrasonography revealed a huge prostatic mass measuring 13×10×9 cm. A magnetic resonance image of the prostate showed an enlarged prostatic mass with hemorrhagic necrosis. The prostatic mass had a large size of 165×99 mm heterogeneous enhancement and displaced the bladder anteriorly and the rectum posteriorly. This implies that the tumor was mainly localized within the prostate, and there was no definite evidence of the direct invasion of adjacent organs (Fig. 1). Abdominal, pelvic, and thoracic computed tomography scans showed a heterogeneous, infiltrative tumor within the prostate gland extending to the bladder and the rectum, no lymph node involvement or any metastatic focus including bones was recorded.

**Figure 2:**
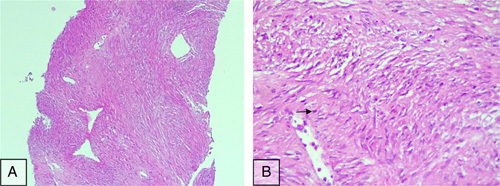
Photomicrographs of hematoxylin and eosin (H&E)-stained sections showing (A) a cellular neoplasm composed of bland spindle cells forming interlacing fascicles with no evidence of necrosis (×100 magnification) and (B) a neoplastic cell with anon-atypical mitotic figure (arrow) (×400 magnification).

A transrectal ultrasound-guided prostate biopsy was performed. Sextant needle core biopsies containing specimens of the prostate have been submitted for histopathological examination. All specimens were fixed in 10% neutral formalin and embedded in paraffin. Three-micron-thick hematoxylin and eosin (H&E) stained sections were obtained. Microscopically, four core biopsies revealed the presence of neoplastic proliferation of spindle-shaped mesenchymal cells forming interlacing fascicles. There were few mitotic figures with a low mitotic count (<1 per 10 high-power fields). There was no evidence of necrosis or nuclear atypia (Fig. 2). The other biopsy cylinders were taken from normal prostate tissue.

**Figure 3:**
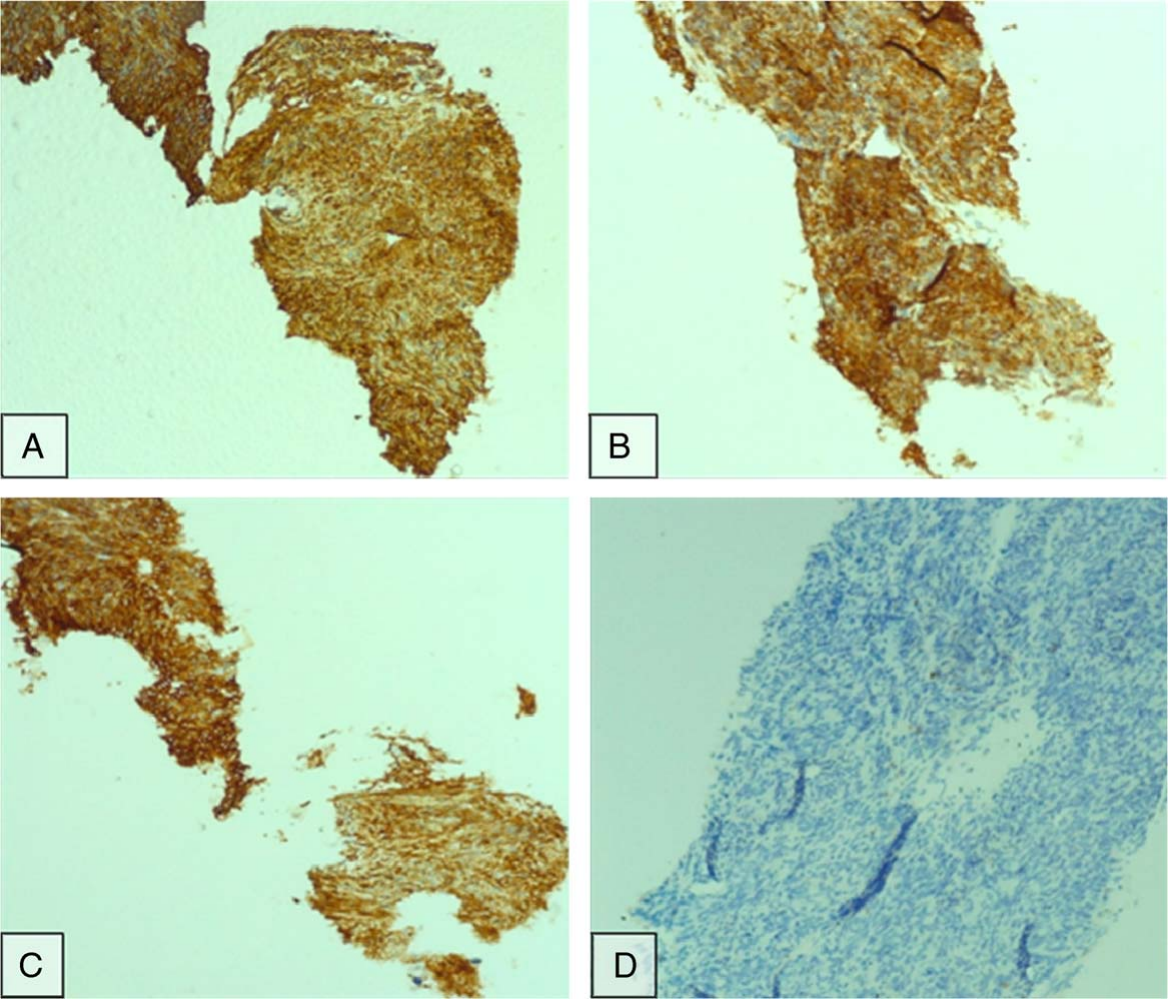
Immunohistochemistry images at ×100 magnification: (A) CD34, (B) CD117, (C) DOG1, and (D) desmin.

In an immunohistochemistry study, the tumor cells were diffusely immunoreactive for CD117, CD34, and DOG1 (Fig. 3).

Thus, the diagnosis of a high-risk primary EGIST of the prostate was made (tumor size >10 cm). The patient refused radical prostatectomy and received only imatinib treatment. Follow-up control was planned after 3 months. The patient was contacted by telephone, and he is in good health with no worsening of symptoms 2 months after his prostate biopsy.

## Discussion

GIST is a nonepithelial, mesenchymal tumor of the gastrointestinal tract, occurring predominantly in the stomach and small and large intestines. A mutation in c-kit exons 9, 11, 13, and 17 and PDGFRA exons 12, 14, and 18 are responsible for the activation of the gene signaling system, which results in uncontrolled phosphorylation and tissue growth^19^. EGISTs can develop on intraperitoneal spaces such as omentum, mesenterium, and gallbladder, and they can occur in retroperitoneum, extraperitoneal (prostate), and intrapelvic organs and share similar histological and immunophenotypic features as GISTs. As a very rare tumor, EGIST constitutes only 5% of GIST^17^. However, EGIST of the prostate is a rare presentation site. The mean patient age at diagnosis is 58 years (range: 31–82)^20^. The clinical presentation is also variable but generally includes voiding LUTS, hematuria, acute urinary retention, perineal pain, or an abnormal rectal examination and a grossly enlarged prostate gland with no elevation in serum PSA levels. Radiologic diagnosis of EGISTs is challenging; MRI and computed tomography (CT) scans are important imaging methods for the diagnosis of EGIST. MRI not only provides information regarding tumor growth and the connection between the tumor and adjacent tissues, but it also contributes to the definition of the hematoma size, necrotic area, and diagnosis of benign from malignant disease^17^. A pathological puncture biopsy can help us identify EGISTs. Among cell types, spindle cells are the most common cell type in EGIST tissues, accounting for about 70% of the number of cells in EGIST tissues. The cells are spiral-shaped, cytoplasmic clumps, reddish; the membrane is not clear, even stained, and the nucleus is not clear. In immunohistochemistry, CD117 is a fabrication from the c-kit proto-oncogene as a transmembrane receptor egg white of tyrosine kinase, and it is one of the extra unique antibodies to diagnose EGISTs. Moreover, as a highly glycosylated type I transmembrane glycoprotein, CD34 can participate in the transport and colonization of hematopoietic stem cells, and its expression rate in EGISTs is 50–70%. The combined detection of CD117 and CD34 can reduce the missed and false positives of EGISTs. In addition, the overall sensitivity of DOG1 in GISTs is as high as 94.4%^21^. DOG1 is strongly expressed on the cell surface of GIST and is rarely expressed in other soft tissue tumors. In our case, the tumor cells were diffusely immunoreactive for CD117, CD34, and DOG1. Surgical resection remains the primary treatment for nonmetastatic EGISTs. Complete removal of the tumor together with invading surrounding organs, as possible, is required. Considering EGISTs are not sensitive to conventional chemotherapy and radiotherapy, imatinib mesylate is proposed as adjuvant therapy for advanced, unresectable, and metastatic cases. As a tyrosine kinase inhibitor of c-kit and PDGFRA, imatinib mesylate has been proven to be an effective therapy for GISTs and EGISTs^13^. Tumor size and mitotic activity have been reported as significant prognostic factors, which should be considered for precise risk stratification and classification. The choice of surgery depends on the tumor size, location, and extent of infiltration. Radical prostatectomy for low-risk and medium-risk resectable tumors and radical prostatectomy+adjuvant/neoadjuvant chemotherapy for medium-risk and high-risk tumors are the recommended lines of management. Conservative treatment with imatinib alone appears to work well for patients who have lost the chance for surgery or who decline surgery^15^.

We conducted a pooled analysis of 16 cases of primary EGIST of the prostate and our case (Table 1).

Therefore, close follow-up is required and must be based on the risk of recurrence following treatment. Limited data are available to predict the malignant potential of prostatic GIST. Imaging follow-up (abdominal and pelvic cavity CT) is considered a possible strategy with which to control the recurrence of prostatic EGIST.

We reported a rare case of primary EGIST of the prostate, and to the best of our knowledge, only 16 cases have been reported in the literature.

Limitations of the case: the diagnosis of EGIST of the prostate is retained only by a prostate biopsy, and we have no surgical specimen because the patient refused surgical treatment.

## Conclusion

The diagnosis of EGIST of the prostate is extremely rare and depends on imaging studies, pathologic results as well as immunohistochemical findings. Radical prostatectomy is the treatment of choice as long as it is feasible, and treatment with imatinib alone remains an option for patients who refuse surgery.

## Ethical approval

Given the nature of the article, a case report, no ethical approval is required.

## Consent

Written informed consent was obtained from the patient for the publication of this case report and accompanying images. A copy of the written consent is available for review by the Editor-in-Chief of this journal on request.

## Sources of funding

This study has not received any funding.

## Author contribution

W.B.: writing – original draft; M.B.O., S.M., and O.B.: writing and editing; I.O. and K.B.: reviewing and editing; W.H. and M.J.: supervision and review.

## Conflicts of interest disclosure

There are no conflicts of interest.

## Guarantor

Wajdi Benabdallah.

## Provenance and peer review

Not commissioned, externally peer-reviewed.
